# 5-(4-Bromo­phen­yl)-2-(3,4-methyl­ene­dioxy­phen­yl)-3-methyl­sulfanyl-1-benzofuran

**DOI:** 10.1107/S1600536809038148

**Published:** 2009-09-26

**Authors:** Hong Dae Choi, Pil Ja Seo, Byeng Wha Son, Uk Lee

**Affiliations:** a Department of Chemistry, Dongeui University, San 24 Kaya-dong Busanjin-gu, Busan 614-714, Republic of Korea; b Department of Chemistry, Pukyong National University, 599-1 Daeyeon 3-dong, Nam-gu, Busan 608-737, Republic of Korea

## Abstract

The title compound, C_22_H_15_BrO_3_S, crystallizes with four mol­ecules in the asymmetric unit. The 4-bromo­phenyl rings are rotated out of the benzofuran planes, with dihedral angles for the four mol­ecules of 20.8 (2), 17.8 (2), 23.5 (4) and 23.9 (4)°. The dihedral angles between the 3,4-methyl­ene­dioxy­phenyl ring and the benzofuran plane are 13.5 (2), 7.1 (2), 18.6 (3) and 14.2 (3)° in the four mol­ecules. The crystal structure is stabilized by weak nonclassical inter­molecular C—H⋯O hydrogen bonds. The crystal structure also exhibits inter­molecular aromatic π–π inter­actions between the benzene and furan rings and between the 4-bromo­phenyl and 3,4-methyl­enedioxy­phenyl rings from mol­ecules of the same type; the centroid–centroid distances are 3.92 (1) and 3.79 (1), 3.91 (1), 3.77 (1) and 3.77 (1), and 3.79 (1) and 3.75 (1)Å in the four mol­ecules.

## Related literature

For the crystal structures of similar 3-methyl­sulfanyl-2-phenyl-1-benzofuran derivatives, see: Choi, Seo *et al.* (2006 ▶); Choi, Woo *et al.* (2006 ▶). For natural products of benzofuran ring systems, see: Akgul & Anil (2003 ▶); von Reuss & König (2004 ▶).
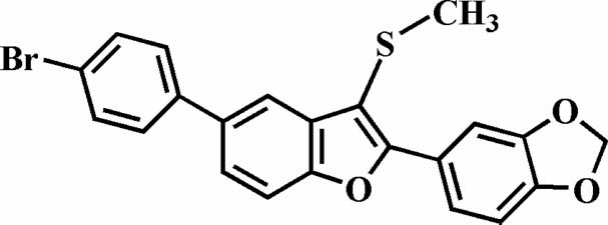


## Experimental

### 

#### Crystal data

C_22_H_15_BrO_3_S
*M* *_r_* = 439.31
Triclinic, 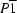

*a* = 12.3757 (8) Å
*b* = 16.067 (1) Å
*c* = 19.587 (1) Å
α = 84.078 (1)°
β = 88.6573 (9)°
γ = 68.0552 (9)°
*V* = 3592.7 (4) Å^3^
*Z* = 8
Mo *K*α radiation
μ = 2.43 mm^−1^
*T* = 173 K
0.24 × 0.16 × 0.12 mm

#### Data collection

Bruker SMART CCD diffractometer
Absorption correction: multi-scan (*SADABS*; Sheldrick, 2000 ▶) *T*_min_ = 0.594, *T*_max_ = 0.760
26887 measured reflections
12562 independent reflections
6573 reflections with *I* > 2σ(*I*)
*R*_int_ = 0.094

#### Refinement

*R*[*F* ^2^ > 2σ(*F* ^2^)] = 0.061
*wR*(*F* ^2^) = 0.150
*S* = 1.06
12562 reflections
973 parameters
H-atom parameters constrained
Δρ_max_ = 0.53 e Å^−3^
Δρ_min_ = −0.73 e Å^−3^

### 

Data collection: *SMART* (Bruker, 2001 ▶); cell refinement: *SAINT* (Bruker, 2001 ▶); data reduction: *SAINT*; program(s) used to solve structure: *SHELXS97* (Sheldrick, 2008 ▶); program(s) used to refine structure: *SHELXL97* (Sheldrick, 2008 ▶); molecular graphics: *ORTEP-3* (Farrugia, 1997 ▶) and *DIAMOND* (Brandenburg, 1998 ▶); software used to prepare material for publication: *SHELXL97*.

## Supplementary Material

Crystal structure: contains datablocks global, I. DOI: 10.1107/S1600536809038148/pb2008sup1.cif

Structure factors: contains datablocks I. DOI: 10.1107/S1600536809038148/pb2008Isup2.hkl

Additional supplementary materials:  crystallographic information; 3D view; checkCIF report

## Figures and Tables

**Table 1:** Hydrogen-bond geometry (Å, °)

*D*—H⋯*A*	*D*—H	H⋯*A*	*D*⋯*A*	*D*—H⋯*A*
C12—H12*A*⋯O4	0.99	2.58	3.50 (1)	154
C21—H21⋯O6^i^	0.95	2.51	3.37 (1)	150
C57—H57*A*⋯O10^ii^	0.99	2.50	3.32 (1)	141
C65—H65⋯O11^iii^	0.95	2.58	3.47 (1)	156
C79—H79*B*⋯O7^iv^	0.99	2.56	3.26 (1)	128

Symmetry codes: (i) 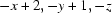; (ii) 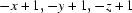; (iii) 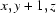; (iv) 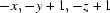.

**Fig. 1.**
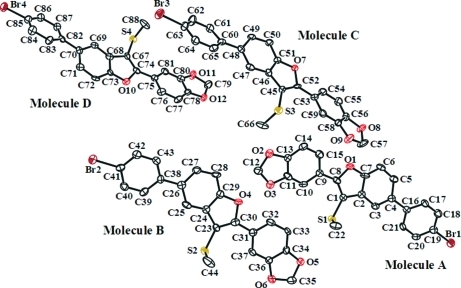
The molecular structure of the title compound with the atom numbering scheme. Displacement ellipsoids are drawn at the 50% probability level. H atoms are presented as a small cycles of arbitrary radius.

**Fig. 2.**
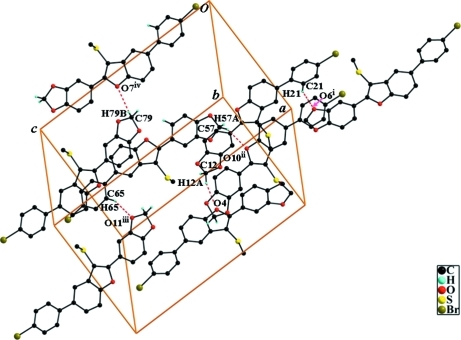
C-H···O hydrogen bonds (dotted lines) in the title compound. [Symmetry codes: (i) - x + 2, - y + 1, - z; (ii) - x + 1, - y + 1, - z + 1; (iii) x, y + 1, z; (iv) - x, - y + 1, - z + 1.]

**Fig. 3.**
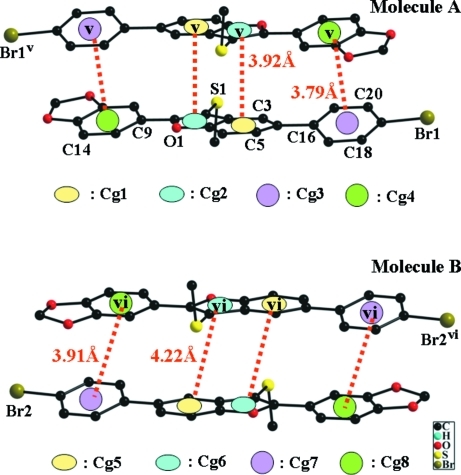
π–π interaction (dotted lines) of molecule A and B in the title compound. Cg denotes the ring centroids. [Symmetry codes: (v) - x + 1, - y + 1, - z; (vi) - x + 2, - y + 1, - z + 1.]

**Fig. 4.**
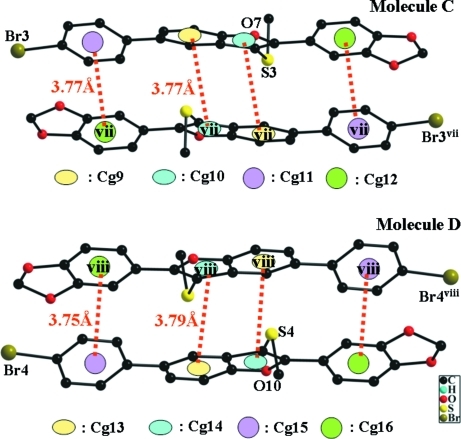
π–π interaction (dotted lines) of molecule C and D in the title compound. Cg denotes the ring centroids. [Symmetry codes: (vii) - x, - y + 2, - z + 1; (viii) - x + 1, - y, - z + 2.]

## supplementary crystallographic information

### Comment

Molecules containing benzofuran skeleton constitute a major group of naturally-occurring compounds that are of considerable interest because of their biological activities (Akgul & Anil, 2003; von Reuss & König, 2004). As a part of our ongoing studies of the effect of side chain substituents on the solid state structures of 3-methylsulfanyl-2-phenyl-1-benzofuran analogues  (Choi, Seo *et al.*, 2006; Choi, Woo *et al.*, 2006), we report the crystal structure of the title compound, which has four unique molecules in the asymmetric unit (further marked as A, B, C and D) (Fig. 1).

The benzofuran unit is essentially planar, with a mean deviation of 0.018 (6) Å for A, 0.011 (6) Å for B, 0.010 (6) Å for C and 0.022 (6) Å for D, respectively, from the least-squares plane defined by the nine constituent atoms. The 4-bromophenyl rings are rotated out of the benzofuran planes, with dihedral angles of 20.8 (2), 6.7 (2), 23.5 (4) and 23.9 (4)° in the molecules A, B, C and D, respectively. The dihedral angles between the 3,4-methylenedioxyphenyl ring and the benzofuran plane are 13.5 (2), 7.1 (2), 18.6 (3) and 14.2 (3)° for the molecules A, B, C and D, respectively.

The molecular packing (Fig. 2) is stabilized by weak non-classical C–H···O hydrogen bonds; the first between the methylene H atom and the furan O atom, with a C12–H12A···O4, the second between the 4-bromophenyl H atom and the oxygen of the methylenedioxy group, with a C21–H21···O6^i^, the third between the methylene H atom and the furan O atom, with a C57–H57A···O10^ii^, the fourth between the 4-bromophenyl H atom and the oxygen of the methylenedioxy group, with a C65–H65···O11^iii^, the fifth between the methylene H atom and the furan O atom, with a C79–H79B···O7^iv^, respectively (Table 1).

The crystal packing (Fig. 3) exhibits aromatic π–π interactions between the benzene/the furan rings and the 4-bromophenyl/the 3,4-methylenedioxyphenyl rings from two A molecules [Cg1···Cg2^v^ = 3.92 (1) Å, Cg3···Cg4^v^ = 3.79 (1) Å] (Cg1, Cg2, Cg3 and Cg4 are the centroids of the C2-C7 benzene ring, the C1/C2/C7/O1/C8 furan ring, the C16-C21 phenyl ring and the C9/C10/C11/C13/C14/C15 phenyl ring, respectively). The crystal packing (Fig. 3) also exhibits aromatic π–π interactions between the benzene/the furan rings and the 4-bromophenyl/the 3,4-methylenedioxyphenyl rings from two B molecules [Cg5···Cg6^vi^ = 4.22 (1) Å, Cg7···Cg8^vi^ = 3.91 (1) Å] (Cg5, Cg6, Cg7 and Cg8 are the centroids of the C24-C29 benzene ring, the C23/C24/C29/O4/C30 furan ring, the C38-C43 phenyl ring and the C31/C32/C33/C34/C36/C37 phenyl ring, respectively). The crystal packing (Fig. 4) exhibits aromatic π–π interactions between the benzene/the furan rings and the 4-bromophenyl/the 3,4-methylenedioxyphenyl rings from two C molecules [Cg9···Cg10^vii^ = 3.77 (1) Å, Cg11···Cg12^vii^ = 3.77 (1) Å] (Cg9, Cg10, Cg11 and Cg12 are the centroids of the C46-C51 benzene ring, the C45/C46/C51/O7/C52 furan ring, the C60-C65 phenyl ring and the C53/C54/C55/C56/C58/C59 phenyl ring, respectively). The crystal packing (Fig. 4) exhibits aromatic π–π interactions between the benzene/the furan rings and the 4-bromophenyl/the 3,4-methylenedioxyphenyl rings from two D molecules [Cg13···Cg14^viii^ = 3.79 (1) Å, Cg15···Cg16^viii^ = 3.75 (1) Å] (Cg13, Cg14, Cg15 and Cg16 are the centroids of the C68-C73 benzene ring, the C67/C68/C73/O10/C74 furan ring, the C82-C87 phenyl ring and the C75/C76/C77/C78/C80/C81 phenyl ring, respectively).

### Experimental

Zinc chloride (409 mg, 3.0 mmol) was added to a stirred solution of 4'-bromo-[1,1'-biphenyl]-4-ol (747 mg, 3.0 mmol) and 2-chloro-2-methylsulfanyl-(3',4'-methylenedioxy)acetophenone (733 mg, 3.0 mmol) in dichloromethane (40 ml) at room temperature, and stirring was continued at the same temperature for 40 min. The reaction was quenched by the addition of water and the organic layer separated, dried over magnesium sulfate, filtered and concentrated in vacuum. The residue was purified by column chromatography (carbon tetrachloride) to afford the title compound as a colorless solid [yield 62%, m.p. 456-457 K; *R*_f_ = 0.35 (carbon tetrachloride)]. Single crystals suitable for X-ray diffraction were prepared by evaporation of a solution of the title compound in tetrahydrofuran at room temperature. Spectroscopic analysis: ^1^H NMR ( CDCl_3_, 400 MHz) δ 2.38 (s, 3H), 6.04 (s, 2H), 6.94 (d, J = 8.6 Hz, 1H), 7.49 (d, J = 1.96 Hz, 1H), 7.50-7.54 (m, 3H), 7.56-7.59 (m, 2H), 7.81 (d, J = 1.56 Hz, 1H), 7.84-7.87 (m, 2H); ^13^C NMR (CDCl_3_, 100 MHz) δ 18.4, 101.4, 107.5, 107.9, 108.5, 111.4, 117.9, 121.2, 121.9, 124.1, 124.2, 129.1, 131.8, 131.9, 135.6, 140.4, 147.8, 148.4, 153.1, 156.1; EI-MS 438 [M^+^], 440 [M+2]

### Refinement

All H atoms were geometrically positioned and refined using a riding model, with C–H = 0.95 Å for the aryl, 0.99 Å for the methylene, and 0.98 Å for the methyl H atoms. *U*iso(H) = 1.2*U*_eq_(C) for the aryl and methylene H atoms, and 1.5*U*_eq_(C) for methyl H atoms.

### Crystal data

**Table:**

C_22_H_15_BrO_3_S	*Z* = 8
*M**_r_* = 439.31	*F*(000) = 1776
Triclinic, *P*1	*D*_x_ = 1.624 Mg m^−^^3^
Hall symbol: -P 1	Mo *K*α radiation, λ = 0.71073 Å
*a* = 12.3757 (8) Å	Cell parameters from 3903 reflections
*b* = 16.067 (1) Å	θ = 2.7–22.5°
*c* = 19.587 (1) Å	µ = 2.42 mm^−^^1^
α = 84.078 (1)°	*T* = 173 K
β = 88.6573 (9)°	Block, colorless
γ = 68.0552 (9)°	0.24 × 0.16 × 0.12 mm
*V* = 3592.7 (4) Å^3^	

### Data collection

**Table:**

Bruker SMART CCD diffractometer	12562 independent reflections
Radiation source: fine-focus sealed tube	6573 reflections with *I* > 2σ(*I*)
graphite	*R*_int_ = 0.094
Detector resolution: 10.0 pixels mm^-1^	θ_max_ = 25.0°, θ_min_ = 1.1°
φ and ω scans	*h* = −14→14
Absorption correction: multi-scan (*SADABS*; Sheldrick, 2000)	*k* = −19→19
*T*_min_ = 0.594, *T*_max_ = 0.760	*l* = −23→23
26887 measured reflections	

### Refinement

**Table:**

Refinement on *F*^2^	Primary atom site location: structure-invariant direct methods
Least-squares matrix: full	Secondary atom site location: difference Fourier map
*R*[*F*^2^ > 2σ(*F*^2^)] = 0.061	Hydrogen site location: difference Fourier map
*wR*(*F*^2^) = 0.150	H-atom parameters constrained
*S* = 1.06	*w* = 1/[σ^2^(*F*_o_^2^) + 15.1547*P*] where *P* = (*F*_o_^2^ + 2*F*_c_^2^)/3
12562 reflections	(Δ/σ)_max_ < 0.001
973 parameters	Δρ_max_ = 0.53 e Å^−^^3^
0 restraints	Δρ_min_ = −0.73 e Å^−^^3^

### Special details

**Table:**

Geometry. All esds (except the esd in the dihedral angle between two l.s. planes) are estimated using the full covariance matrix. The cell esds are taken into account individually in the estimation of esds in distances, angles and torsion angles; correlations between esds in cell parameters are only used when they are defined by crystal symmetry. An approximate (isotropic) treatment of cell esds is used for estimating esds involving l.s. planes.
Refinement. Refinement of F^2^ against ALL reflections. The weighted R-factor wR and goodness of fit S are based on F^2^, conventional R-factors R are based on F, with F set to zero for negative F^2^. The threshold expression of F^2^ > 2sigma(F^2^) is used only for calculating R-factors(gt) etc. and is not relevant to the choice of reflections for refinement. R-factors based on F^2^ are statistically about twice as large as those based on F, and R- factors based on ALL data will be even larger.

### Fractional atomic coordinates and isotropic or equivalent isotropic displacement parameters (Å^2^)

**Table:**

	*x*	*y*	*z*	*U*_iso_*/*U*_eq_	
Br1	0.50384 (8)	0.59231 (7)	−0.45348 (5)	0.0387 (3)	
Br2	0.98757 (8)	0.61526 (7)	0.94332 (5)	0.0377 (3)	
Br3	0.08943 (8)	0.90050 (7)	0.95449 (5)	0.0389 (3)	
Br4	0.42898 (8)	0.09093 (7)	1.43447 (4)	0.0381 (3)	
S1	0.70263 (18)	0.55819 (14)	0.03947 (11)	0.0276 (5)	
O1	0.3585 (4)	0.6481 (4)	0.0621 (3)	0.0292 (14)	
O2	0.5119 (5)	0.6257 (4)	0.3701 (3)	0.0353 (15)	
O3	0.6786 (5)	0.5523 (4)	0.3112 (3)	0.0407 (16)	
C1	0.5504 (7)	0.5988 (5)	0.0350 (4)	0.0208 (19)	
C2	0.4815 (7)	0.6134 (5)	−0.0271 (4)	0.023 (2)	
C3	0.5089 (7)	0.6079 (5)	−0.0969 (4)	0.025 (2)	
H3	0.5876	0.5893	−0.1111	0.030*	
C4	0.4193 (7)	0.6301 (5)	−0.1448 (4)	0.025 (2)	
C5	0.3025 (7)	0.6563 (5)	−0.1211 (4)	0.029 (2)	
H5	0.2415	0.6683	−0.1538	0.035*	
C6	0.2738 (7)	0.6649 (5)	−0.0528 (4)	0.029 (2)	
H6	0.1954	0.6845	−0.0379	0.035*	
C7	0.3664 (7)	0.6433 (6)	−0.0078 (4)	0.029 (2)	
C8	0.4726 (7)	0.6201 (5)	0.0877 (4)	0.027 (2)	
C9	0.4809 (7)	0.6233 (5)	0.1615 (4)	0.024 (2)	
C10	0.5869 (7)	0.5820 (6)	0.1977 (4)	0.027 (2)	
H10	0.6572	0.5514	0.1750	0.032*	
C11	0.5854 (7)	0.5875 (6)	0.2657 (5)	0.030 (2)	
C12	0.6349 (7)	0.5768 (7)	0.3773 (5)	0.043 (3)	
H12A	0.6729	0.6148	0.3950	0.051*	
H12B	0.6513	0.5221	0.4100	0.051*	
C13	0.4852 (7)	0.6319 (5)	0.3001 (4)	0.0230 (19)	
C14	0.3804 (8)	0.6730 (6)	0.2674 (4)	0.032 (2)	
H14	0.3120	0.7044	0.2913	0.038*	
C15	0.3779 (7)	0.6670 (6)	0.1973 (4)	0.032 (2)	
H15	0.3056	0.6928	0.1729	0.039*	
C16	0.4390 (7)	0.6245 (5)	−0.2201 (4)	0.0224 (19)	
C17	0.3524 (7)	0.6734 (6)	−0.2691 (4)	0.028 (2)	
H17	0.2794	0.7136	−0.2547	0.034*	
C18	0.3712 (7)	0.6640 (6)	−0.3376 (5)	0.034 (2)	
H18	0.3101	0.6952	−0.3701	0.040*	
C19	0.4777 (7)	0.6098 (6)	−0.3593 (4)	0.031 (2)	
C20	0.5679 (7)	0.5649 (5)	−0.3125 (4)	0.028 (2)	
H20	0.6430	0.5297	−0.3278	0.034*	
C21	0.5474 (7)	0.5717 (5)	−0.2431 (4)	0.028 (2)	
H21	0.6086	0.5397	−0.2108	0.033*	
C22	0.7251 (7)	0.6617 (6)	0.0456 (5)	0.040 (2)	
H22A	0.8088	0.6491	0.0484	0.061*	
H22B	0.6873	0.6886	0.0869	0.061*	
H22C	0.6913	0.7038	0.0049	0.061*	
S2	1.19277 (18)	0.55578 (15)	0.45491 (11)	0.0297 (5)	
O4	0.8489 (4)	0.6558 (4)	0.4222 (3)	0.0278 (14)	
O5	1.0111 (5)	0.6096 (4)	0.1168 (3)	0.0326 (15)	
O6	1.1794 (5)	0.5482 (4)	0.1839 (3)	0.0402 (16)	
C23	1.0399 (7)	0.6017 (5)	0.4537 (4)	0.0224 (19)	
C24	0.9693 (7)	0.6214 (5)	0.5151 (4)	0.0232 (19)	
C25	0.9937 (7)	0.6146 (5)	0.5844 (4)	0.028 (2)	
H25	1.0722	0.5932	0.6004	0.034*	
C26	0.9024 (7)	0.6393 (5)	0.6310 (4)	0.0235 (19)	
C27	0.7873 (7)	0.6691 (6)	0.6048 (4)	0.031 (2)	
H27	0.7250	0.6841	0.6362	0.037*	
C28	0.7610 (7)	0.6775 (6)	0.5356 (4)	0.032 (2)	
H28	0.6830	0.6988	0.5188	0.038*	
C29	0.8547 (7)	0.6531 (5)	0.4927 (4)	0.026 (2)	
C30	0.9640 (7)	0.6234 (5)	0.4002 (4)	0.026 (2)	
C31	0.9733 (7)	0.6229 (5)	0.3258 (4)	0.025 (2)	
C32	0.8737 (7)	0.6596 (6)	0.2840 (5)	0.031 (2)	
H32	0.8002	0.6862	0.3048	0.038*	
C33	0.8777 (7)	0.6586 (6)	0.2127 (4)	0.028 (2)	
H33	0.8089	0.6830	0.1849	0.034*	
C34	0.9845 (7)	0.6211 (5)	0.1851 (4)	0.026 (2)	
C35	1.1354 (7)	0.5715 (7)	0.1157 (5)	0.045 (3)	
H35A	1.1615	0.5171	0.0906	0.054*	
H35B	1.1654	0.6157	0.0918	0.054*	
C36	1.0832 (7)	0.5839 (5)	0.2255 (4)	0.026 (2)	
C37	1.0819 (7)	0.5830 (5)	0.2946 (4)	0.027 (2)	
H37	1.1518	0.5563	0.3212	0.032*	
C38	0.9236 (7)	0.6350 (5)	0.7062 (4)	0.0220 (19)	
C39	1.0321 (7)	0.5840 (6)	0.7365 (4)	0.029 (2)	
H39	1.0943	0.5521	0.7083	0.034*	
C40	1.0520 (7)	0.5785 (5)	0.8059 (4)	0.029 (2)	
H40	1.1269	0.5432	0.8251	0.035*	
C41	0.9627 (7)	0.6243 (5)	0.8475 (4)	0.025 (2)	
C42	0.8536 (7)	0.6781 (6)	0.8188 (4)	0.034 (2)	
H42	0.7920	0.7109	0.8469	0.041*	
C43	0.8366 (7)	0.6828 (6)	0.7496 (4)	0.032 (2)	
H43	0.7625	0.7202	0.7303	0.038*	
C44	1.2242 (8)	0.6572 (6)	0.4457 (6)	0.054 (3)	
H44A	1.3088	0.6412	0.4456	0.082*	
H44B	1.1907	0.6928	0.4841	0.082*	
H44C	1.1903	0.6928	0.4024	0.082*	
S3	0.26939 (18)	0.93222 (15)	0.45490 (11)	0.0268 (5)	
O7	−0.0074 (4)	0.8653 (3)	0.4399 (3)	0.0220 (13)	
O8	0.1301 (5)	0.8741 (4)	0.1293 (3)	0.0356 (15)	
O9	0.2265 (5)	0.9503 (4)	0.1814 (3)	0.0399 (16)	
C45	0.1473 (6)	0.9008 (5)	0.4627 (4)	0.0184 (18)	
C46	0.0944 (7)	0.8877 (5)	0.5277 (4)	0.0205 (19)	
C47	0.1165 (7)	0.8896 (5)	0.5959 (4)	0.024 (2)	
H47	0.1808	0.9036	0.6089	0.028*	
C48	0.0439 (6)	0.8710 (5)	0.6462 (4)	0.0199 (18)	
C49	−0.0528 (6)	0.8520 (5)	0.6248 (4)	0.0212 (19)	
H49	−0.1038	0.8413	0.6585	0.025*	
C50	−0.0743 (7)	0.8485 (5)	0.5572 (4)	0.024 (2)	
H50	−0.1386	0.8348	0.5437	0.029*	
C51	0.0002 (7)	0.8655 (5)	0.5095 (4)	0.025 (2)	
C52	0.0862 (6)	0.8874 (5)	0.4125 (4)	0.0212 (19)	
C53	0.0951 (6)	0.8823 (5)	0.3379 (4)	0.0206 (19)	
C54	0.0372 (7)	0.8371 (5)	0.3053 (4)	0.025 (2)	
H54	−0.0083	0.8100	0.3320	0.030*	
C55	0.0443 (7)	0.8308 (6)	0.2350 (4)	0.029 (2)	
H55	0.0041	0.8006	0.2131	0.034*	
C56	0.1115 (7)	0.8699 (5)	0.1987 (4)	0.024 (2)	
C57	0.2121 (8)	0.9185 (8)	0.1186 (4)	0.050 (3)	
H57A	0.2878	0.8757	0.1040	0.060*	
H57B	0.1825	0.9696	0.0823	0.060*	
C58	0.1688 (7)	0.9152 (5)	0.2306 (4)	0.0226 (19)	
C59	0.1631 (7)	0.9225 (5)	0.2981 (4)	0.026 (2)	
H59	0.2035	0.9537	0.3187	0.031*	
C60	0.0611 (6)	0.8750 (5)	0.7200 (4)	0.0212 (19)	
C61	0.0161 (7)	0.8278 (5)	0.7701 (4)	0.026 (2)	
H61	−0.0217	0.7905	0.7562	0.032*	
C62	0.0257 (8)	0.8349 (6)	0.8384 (4)	0.035 (2)	
H62	−0.0073	0.8039	0.8713	0.042*	
C63	0.0827 (7)	0.8866 (6)	0.8599 (4)	0.027 (2)	
C64	0.1315 (7)	0.9312 (6)	0.8122 (4)	0.028 (2)	
H64	0.1726	0.9657	0.8269	0.034*	
C65	0.1204 (7)	0.9255 (5)	0.7438 (4)	0.026 (2)	
H65	0.1540	0.9567	0.7115	0.031*	
C66	0.3844 (7)	0.8217 (6)	0.4697 (5)	0.045 (3)	
H66A	0.4603	0.8278	0.4669	0.068*	
H66B	0.3790	0.7838	0.4347	0.068*	
H66C	0.3761	0.7937	0.5153	0.068*	
S4	0.23241 (18)	0.06678 (15)	0.94641 (11)	0.0272 (5)	
O10	0.5046 (4)	0.1399 (4)	0.9101 (3)	0.0260 (13)	
O11	0.2639 (5)	0.0615 (4)	0.6721 (3)	0.0390 (16)	
O12	0.3684 (5)	0.1314 (4)	0.6048 (3)	0.0318 (15)	
C67	0.3528 (6)	0.1008 (5)	0.9437 (4)	0.0207 (19)	
C68	0.4092 (7)	0.1112 (5)	1.0048 (4)	0.024 (2)	
C69	0.3869 (7)	0.1089 (5)	1.0745 (4)	0.0231 (19)	
H69	0.3228	0.0949	1.0920	0.028*	
C70	0.4605 (7)	0.1275 (5)	1.1188 (4)	0.0232 (19)	
C71	0.5530 (7)	0.1510 (5)	1.0910 (4)	0.025 (2)	
H71	0.6023	0.1638	1.1212	0.030*	
C72	0.5741 (6)	0.1559 (5)	1.0216 (4)	0.027 (2)	
H72	0.6364	0.1718	1.0034	0.032*	
C73	0.4992 (6)	0.1362 (5)	0.9795 (4)	0.0209 (19)	
C74	0.4106 (7)	0.1194 (5)	0.8889 (4)	0.024 (2)	
C75	0.4005 (7)	0.1234 (5)	0.8139 (4)	0.0231 (19)	
C76	0.4619 (7)	0.1663 (6)	0.7723 (4)	0.031 (2)	
H76	0.5087	0.1919	0.7934	0.037*	
C77	0.4563 (7)	0.1724 (5)	0.7009 (4)	0.029 (2)	
H77	0.4998	0.2001	0.6728	0.035*	
C78	0.3859 (7)	0.1370 (5)	0.6735 (4)	0.025 (2)	
C79	0.2758 (8)	0.0978 (7)	0.6031 (4)	0.041 (2)	
H79A	0.2950	0.0502	0.5715	0.049*	
H79B	0.2022	0.1471	0.5871	0.049*	
C80	0.3247 (7)	0.0941 (5)	0.7139 (4)	0.026 (2)	
C81	0.3293 (7)	0.0861 (5)	0.7832 (4)	0.028 (2)	
H81	0.2864	0.0568	0.8101	0.033*	
C82	0.4455 (6)	0.1239 (5)	1.1945 (4)	0.0208 (19)	
C83	0.4896 (7)	0.1713 (6)	1.2348 (4)	0.030 (2)	
H83	0.5261	0.2093	1.2129	0.036*	
C84	0.4817 (7)	0.1646 (6)	1.3051 (4)	0.033 (2)	
H84	0.5108	0.1983	1.3314	0.039*	
C85	0.4309 (7)	0.1082 (6)	1.3366 (4)	0.027 (2)	
C86	0.3821 (7)	0.0630 (5)	1.2990 (4)	0.028 (2)	
H86	0.3441	0.0265	1.3214	0.034*	
C87	0.3887 (7)	0.0711 (5)	1.2290 (4)	0.026 (2)	
H87	0.3542	0.0404	1.2032	0.031*	
C88	0.1155 (7)	0.1741 (6)	0.9358 (5)	0.048 (3)	
H88A	0.0411	0.1655	0.9363	0.072*	
H88B	0.1231	0.2065	0.8919	0.072*	
H88C	0.1180	0.2092	0.9734	0.072*	

### Atomic displacement parameters (Å^2^)

**Table:**

	*U*^11^	*U*^22^	*U*^33^	*U*^12^	*U*^13^	*U*^23^
Br1	0.0384 (6)	0.0440 (6)	0.0303 (5)	−0.0108 (5)	0.0001 (4)	−0.0061 (5)
Br2	0.0373 (6)	0.0498 (6)	0.0261 (5)	−0.0166 (5)	0.0002 (4)	−0.0027 (5)
Br3	0.0489 (6)	0.0550 (7)	0.0248 (5)	−0.0313 (5)	0.0044 (5)	−0.0127 (5)
Br4	0.0449 (6)	0.0502 (6)	0.0241 (5)	−0.0239 (5)	−0.0008 (4)	−0.0008 (4)
S1	0.0227 (12)	0.0264 (13)	0.0307 (13)	−0.0048 (10)	0.0014 (10)	−0.0068 (10)
O1	0.023 (3)	0.042 (4)	0.028 (3)	−0.018 (3)	0.003 (3)	−0.006 (3)
O2	0.037 (4)	0.046 (4)	0.025 (3)	−0.017 (3)	0.002 (3)	−0.007 (3)
O3	0.034 (4)	0.061 (5)	0.028 (4)	−0.018 (3)	0.000 (3)	−0.003 (3)
C1	0.024 (5)	0.016 (4)	0.026 (5)	−0.010 (4)	−0.001 (4)	−0.004 (4)
C2	0.030 (5)	0.014 (5)	0.026 (5)	−0.007 (4)	0.004 (4)	−0.007 (4)
C3	0.023 (5)	0.015 (5)	0.032 (5)	−0.001 (4)	0.006 (4)	−0.006 (4)
C4	0.026 (5)	0.020 (5)	0.030 (5)	−0.009 (4)	0.003 (4)	−0.004 (4)
C5	0.024 (5)	0.030 (5)	0.036 (6)	−0.012 (4)	0.000 (4)	−0.008 (4)
C6	0.017 (5)	0.028 (5)	0.045 (6)	−0.010 (4)	0.000 (4)	−0.008 (4)
C7	0.015 (5)	0.035 (5)	0.039 (6)	−0.010 (4)	0.009 (4)	−0.016 (4)
C8	0.019 (5)	0.027 (5)	0.037 (6)	−0.010 (4)	−0.004 (4)	−0.009 (4)
C9	0.025 (5)	0.021 (5)	0.027 (5)	−0.008 (4)	0.000 (4)	−0.003 (4)
C10	0.021 (5)	0.033 (5)	0.030 (5)	−0.013 (4)	0.010 (4)	−0.011 (4)
C11	0.022 (5)	0.026 (5)	0.043 (6)	−0.012 (4)	−0.005 (4)	−0.002 (4)
C12	0.028 (5)	0.067 (7)	0.034 (6)	−0.021 (5)	0.001 (5)	−0.004 (5)
C13	0.026 (5)	0.022 (5)	0.023 (5)	−0.010 (4)	0.001 (4)	−0.004 (4)
C14	0.035 (5)	0.038 (6)	0.026 (5)	−0.016 (5)	0.008 (4)	−0.009 (4)
C15	0.029 (5)	0.028 (5)	0.036 (6)	−0.007 (4)	0.000 (4)	−0.005 (4)
C16	0.023 (5)	0.019 (5)	0.030 (5)	−0.013 (4)	−0.002 (4)	−0.005 (4)
C17	0.024 (5)	0.031 (5)	0.028 (5)	−0.006 (4)	−0.005 (4)	−0.007 (4)
C18	0.030 (5)	0.032 (6)	0.034 (6)	−0.006 (4)	−0.001 (4)	−0.007 (4)
C19	0.031 (5)	0.027 (5)	0.034 (5)	−0.010 (4)	0.003 (4)	−0.005 (4)
C20	0.023 (5)	0.027 (5)	0.036 (5)	−0.011 (4)	0.008 (4)	−0.006 (4)
C21	0.024 (5)	0.029 (5)	0.026 (5)	−0.005 (4)	−0.007 (4)	0.002 (4)
C22	0.029 (5)	0.040 (6)	0.065 (7)	−0.025 (5)	0.008 (5)	−0.016 (5)
S2	0.0232 (12)	0.0299 (13)	0.0319 (13)	−0.0052 (10)	−0.0029 (10)	−0.0023 (10)
O4	0.019 (3)	0.039 (4)	0.030 (3)	−0.016 (3)	0.005 (3)	−0.007 (3)
O5	0.025 (3)	0.039 (4)	0.026 (3)	−0.004 (3)	0.001 (3)	−0.001 (3)
O6	0.025 (3)	0.056 (4)	0.027 (4)	0.000 (3)	0.000 (3)	−0.010 (3)
C23	0.022 (5)	0.021 (5)	0.026 (5)	−0.009 (4)	−0.005 (4)	−0.004 (4)
C24	0.028 (5)	0.021 (5)	0.024 (5)	−0.011 (4)	0.001 (4)	−0.007 (4)
C25	0.019 (5)	0.025 (5)	0.045 (6)	−0.013 (4)	−0.002 (4)	−0.001 (4)
C26	0.029 (5)	0.010 (4)	0.031 (5)	−0.007 (4)	0.003 (4)	−0.003 (4)
C27	0.026 (5)	0.045 (6)	0.029 (5)	−0.020 (4)	0.003 (4)	−0.009 (4)
C28	0.018 (5)	0.048 (6)	0.035 (6)	−0.020 (4)	0.006 (4)	−0.006 (5)
C29	0.031 (5)	0.027 (5)	0.024 (5)	−0.017 (4)	−0.001 (4)	−0.003 (4)
C30	0.028 (5)	0.021 (5)	0.031 (5)	−0.010 (4)	0.008 (4)	−0.008 (4)
C31	0.027 (5)	0.019 (5)	0.029 (5)	−0.009 (4)	0.002 (4)	0.000 (4)
C32	0.018 (5)	0.030 (5)	0.043 (6)	−0.006 (4)	0.001 (4)	−0.001 (4)
C33	0.016 (5)	0.040 (6)	0.024 (5)	−0.005 (4)	−0.007 (4)	0.001 (4)
C34	0.030 (5)	0.030 (5)	0.016 (5)	−0.009 (4)	−0.001 (4)	−0.008 (4)
C35	0.028 (5)	0.069 (7)	0.035 (6)	−0.014 (5)	0.008 (5)	−0.006 (5)
C36	0.022 (5)	0.026 (5)	0.026 (5)	−0.005 (4)	0.001 (4)	0.001 (4)
C37	0.018 (5)	0.028 (5)	0.031 (5)	−0.004 (4)	−0.005 (4)	−0.004 (4)
C38	0.029 (5)	0.020 (5)	0.021 (5)	−0.015 (4)	−0.005 (4)	0.004 (4)
C39	0.038 (5)	0.028 (5)	0.024 (5)	−0.014 (4)	0.008 (4)	−0.008 (4)
C40	0.024 (5)	0.026 (5)	0.034 (5)	−0.007 (4)	−0.004 (4)	0.004 (4)
C41	0.028 (5)	0.024 (5)	0.029 (5)	−0.014 (4)	0.007 (4)	−0.005 (4)
C42	0.028 (5)	0.037 (6)	0.032 (6)	−0.007 (4)	0.002 (4)	−0.001 (4)
C43	0.022 (5)	0.039 (6)	0.029 (5)	−0.007 (4)	−0.002 (4)	0.004 (4)
C44	0.030 (6)	0.042 (7)	0.092 (9)	−0.017 (5)	−0.017 (6)	0.011 (6)
S3	0.0297 (12)	0.0319 (13)	0.0274 (12)	−0.0214 (11)	0.0014 (10)	−0.0031 (10)
O7	0.017 (3)	0.031 (3)	0.020 (3)	−0.013 (3)	0.000 (2)	−0.001 (3)
O8	0.040 (4)	0.056 (4)	0.018 (3)	−0.024 (3)	0.010 (3)	−0.012 (3)
O9	0.048 (4)	0.065 (5)	0.023 (3)	−0.039 (4)	0.006 (3)	−0.008 (3)
C45	0.022 (4)	0.017 (4)	0.017 (4)	−0.009 (4)	0.001 (4)	−0.002 (3)
C46	0.021 (5)	0.018 (5)	0.023 (5)	−0.008 (4)	−0.004 (4)	−0.002 (4)
C47	0.017 (4)	0.023 (5)	0.034 (5)	−0.011 (4)	−0.001 (4)	−0.008 (4)
C48	0.019 (4)	0.013 (4)	0.028 (5)	−0.005 (4)	−0.003 (4)	0.000 (4)
C49	0.016 (4)	0.022 (5)	0.030 (5)	−0.013 (4)	0.005 (4)	0.001 (4)
C50	0.021 (5)	0.032 (5)	0.025 (5)	−0.016 (4)	0.002 (4)	−0.005 (4)
C51	0.021 (5)	0.028 (5)	0.021 (5)	−0.005 (4)	0.003 (4)	−0.004 (4)
C52	0.016 (4)	0.017 (5)	0.028 (5)	−0.005 (4)	0.011 (4)	0.005 (4)
C53	0.020 (4)	0.021 (5)	0.023 (5)	−0.009 (4)	0.004 (4)	−0.005 (4)
C54	0.023 (5)	0.030 (5)	0.028 (5)	−0.016 (4)	0.001 (4)	−0.005 (4)
C55	0.031 (5)	0.031 (5)	0.029 (5)	−0.016 (4)	−0.004 (4)	−0.006 (4)
C56	0.031 (5)	0.021 (5)	0.019 (5)	−0.008 (4)	−0.001 (4)	−0.002 (4)
C57	0.045 (6)	0.097 (9)	0.022 (5)	−0.040 (6)	0.016 (5)	−0.019 (5)
C58	0.023 (5)	0.027 (5)	0.020 (5)	−0.012 (4)	0.000 (4)	−0.001 (4)
C59	0.021 (5)	0.031 (5)	0.027 (5)	−0.011 (4)	−0.004 (4)	−0.004 (4)
C60	0.019 (4)	0.015 (5)	0.029 (5)	−0.007 (4)	0.006 (4)	0.001 (4)
C61	0.027 (5)	0.034 (5)	0.028 (5)	−0.022 (4)	−0.005 (4)	−0.004 (4)
C62	0.049 (6)	0.042 (6)	0.025 (5)	−0.031 (5)	−0.002 (5)	0.005 (4)
C63	0.034 (5)	0.030 (5)	0.020 (5)	−0.015 (4)	−0.001 (4)	−0.007 (4)
C64	0.028 (5)	0.036 (6)	0.028 (5)	−0.020 (4)	−0.005 (4)	−0.004 (4)
C65	0.027 (5)	0.028 (5)	0.028 (5)	−0.016 (4)	0.006 (4)	−0.005 (4)
C66	0.017 (5)	0.044 (6)	0.076 (8)	−0.012 (5)	0.008 (5)	−0.013 (5)
S4	0.0304 (12)	0.0308 (13)	0.0290 (12)	−0.0212 (11)	0.0007 (10)	−0.0028 (10)
O10	0.028 (3)	0.033 (4)	0.025 (3)	−0.019 (3)	0.004 (3)	−0.009 (3)
O11	0.049 (4)	0.061 (5)	0.022 (3)	−0.038 (4)	−0.003 (3)	−0.002 (3)
O12	0.034 (4)	0.051 (4)	0.018 (3)	−0.026 (3)	−0.001 (3)	−0.003 (3)
C67	0.020 (4)	0.018 (5)	0.021 (5)	−0.004 (4)	−0.004 (4)	−0.003 (4)
C68	0.021 (5)	0.016 (5)	0.034 (5)	−0.006 (4)	0.003 (4)	0.001 (4)
C69	0.021 (5)	0.016 (5)	0.031 (5)	−0.006 (4)	0.004 (4)	0.000 (4)
C70	0.026 (5)	0.016 (5)	0.026 (5)	−0.006 (4)	−0.006 (4)	−0.001 (4)
C71	0.026 (5)	0.025 (5)	0.031 (5)	−0.014 (4)	0.002 (4)	−0.013 (4)
C72	0.012 (4)	0.034 (5)	0.037 (5)	−0.010 (4)	0.005 (4)	−0.004 (4)
C73	0.016 (4)	0.024 (5)	0.023 (5)	−0.007 (4)	−0.004 (4)	−0.001 (4)
C74	0.017 (4)	0.028 (5)	0.032 (5)	−0.012 (4)	−0.003 (4)	−0.005 (4)
C75	0.022 (5)	0.022 (5)	0.026 (5)	−0.010 (4)	0.000 (4)	−0.004 (4)
C76	0.033 (5)	0.030 (5)	0.034 (5)	−0.017 (4)	0.007 (4)	−0.010 (4)
C77	0.040 (5)	0.032 (5)	0.027 (5)	−0.026 (5)	0.011 (4)	−0.006 (4)
C78	0.024 (5)	0.030 (5)	0.021 (5)	−0.010 (4)	0.005 (4)	−0.007 (4)
C79	0.044 (6)	0.068 (7)	0.027 (5)	−0.036 (6)	0.002 (5)	−0.010 (5)
C80	0.024 (5)	0.021 (5)	0.035 (5)	−0.011 (4)	0.001 (4)	−0.009 (4)
C81	0.022 (5)	0.030 (5)	0.033 (5)	−0.011 (4)	−0.002 (4)	−0.006 (4)
C82	0.018 (4)	0.016 (5)	0.027 (5)	−0.004 (4)	−0.004 (4)	−0.004 (4)
C83	0.040 (5)	0.028 (5)	0.025 (5)	−0.016 (4)	0.004 (4)	−0.005 (4)
C84	0.046 (6)	0.032 (6)	0.032 (5)	−0.029 (5)	0.001 (4)	−0.003 (4)
C85	0.033 (5)	0.030 (5)	0.024 (5)	−0.019 (4)	0.002 (4)	−0.001 (4)
C86	0.034 (5)	0.026 (5)	0.028 (5)	−0.017 (4)	0.001 (4)	0.003 (4)
C87	0.033 (5)	0.019 (5)	0.026 (5)	−0.009 (4)	−0.002 (4)	−0.006 (4)
C88	0.023 (5)	0.040 (6)	0.078 (8)	−0.011 (5)	−0.016 (5)	0.008 (6)

### Geometric parameters (Å, °)

**Table:**

Br1—C19	1.897 (9)	C44—H44B	0.9800
Br2—C41	1.890 (8)	C44—H44C	0.9800
Br3—C63	1.895 (8)	S3—C45	1.763 (8)
Br4—C85	1.909 (8)	S3—C66	1.813 (9)
S1—C1	1.749 (8)	O7—C51	1.370 (9)
S1—C22	1.802 (8)	O7—C52	1.412 (8)
O1—C7	1.378 (9)	O8—C56	1.373 (9)
O1—C8	1.399 (9)	O8—C57	1.443 (10)
O2—C13	1.403 (9)	O9—C58	1.383 (9)
O2—C12	1.431 (9)	O9—C57	1.419 (10)
O3—C11	1.379 (9)	C45—C52	1.337 (10)
O3—C12	1.428 (10)	C45—C46	1.450 (10)
C1—C8	1.376 (10)	C46—C47	1.376 (10)
C1—C2	1.450 (10)	C46—C51	1.403 (10)
C2—C7	1.379 (10)	C47—C48	1.401 (10)
C2—C3	1.405 (11)	C47—H47	0.9500
C3—C4	1.386 (11)	C48—C49	1.423 (10)
C3—H3	0.9500	C48—C60	1.477 (11)
C4—C5	1.427 (10)	C49—C50	1.367 (10)
C4—C16	1.496 (11)	C49—H49	0.9500
C5—C6	1.383 (11)	C50—C51	1.377 (10)
C5—H5	0.9500	C50—H50	0.9500
C6—C7	1.378 (11)	C52—C53	1.471 (10)
C6—H6	0.9500	C53—C54	1.396 (10)
C8—C9	1.458 (11)	C53—C59	1.417 (10)
C9—C10	1.400 (11)	C54—C55	1.389 (11)
C9—C15	1.414 (11)	C54—H54	0.9500
C10—C11	1.343 (11)	C55—C56	1.363 (11)
C10—H10	0.9500	C55—H55	0.9500
C11—C13	1.379 (11)	C56—C58	1.386 (10)
C12—H12A	0.9900	C57—H57A	0.9900
C12—H12B	0.9900	C57—H57B	0.9900
C13—C14	1.357 (11)	C58—C59	1.337 (10)
C14—C15	1.388 (11)	C59—H59	0.9500
C14—H14	0.9500	C60—C65	1.398 (10)
C15—H15	0.9500	C60—C61	1.409 (10)
C16—C21	1.387 (10)	C61—C62	1.366 (11)
C16—C17	1.396 (10)	C61—H61	0.9500
C17—C18	1.371 (11)	C62—C63	1.374 (11)
C17—H17	0.9500	C62—H62	0.9500
C18—C19	1.366 (11)	C63—C64	1.383 (11)
C18—H18	0.9500	C64—C65	1.365 (11)
C19—C20	1.384 (11)	C64—H64	0.9500
C20—C21	1.385 (11)	C65—H65	0.9500
C20—H20	0.9500	C66—H66A	0.9800
C21—H21	0.9500	C66—H66B	0.9800
C22—H22A	0.9800	C66—H66C	0.9800
C22—H22B	0.9800	S4—C67	1.766 (8)
C22—H22C	0.9800	S4—C88	1.785 (9)
S2—C23	1.755 (8)	O10—C73	1.356 (9)
S2—C44	1.803 (9)	O10—C74	1.403 (8)
O4—C29	1.379 (9)	O11—C80	1.386 (9)
O4—C30	1.396 (9)	O11—C79	1.443 (9)
O5—C34	1.385 (9)	O12—C78	1.385 (9)
O5—C35	1.428 (9)	O12—C79	1.439 (9)
O6—C36	1.396 (9)	C67—C74	1.346 (10)
O6—C35	1.414 (10)	C67—C68	1.455 (11)
C23—C30	1.351 (11)	C68—C69	1.387 (11)
C23—C24	1.461 (10)	C68—C73	1.383 (10)
C24—C25	1.382 (11)	C69—C70	1.402 (10)
C24—C29	1.381 (11)	C69—H69	0.9500
C25—C26	1.398 (10)	C70—C71	1.414 (10)
C25—H25	0.9500	C70—C82	1.489 (11)
C26—C27	1.413 (11)	C71—C72	1.377 (11)
C26—C38	1.493 (11)	C71—H71	0.9500
C27—C28	1.382 (11)	C72—C73	1.396 (10)
C27—H27	0.9500	C72—H72	0.9500
C28—C29	1.373 (10)	C74—C75	1.470 (11)
C28—H28	0.9500	C75—C76	1.401 (11)
C30—C31	1.460 (11)	C75—C81	1.410 (10)
C31—C32	1.394 (11)	C76—C77	1.394 (11)
C31—C37	1.410 (10)	C76—H76	0.9500
C32—C33	1.398 (11)	C77—C78	1.351 (10)
C32—H32	0.9500	C77—H77	0.9500
C33—C34	1.357 (10)	C78—C80	1.387 (11)
C33—H33	0.9500	C79—H79A	0.9900
C34—C36	1.370 (10)	C79—H79B	0.9900
C35—H35A	0.9900	C80—C81	1.351 (11)
C35—H35B	0.9900	C81—H81	0.9500
C36—C37	1.351 (11)	C82—C87	1.405 (10)
C37—H37	0.9500	C82—C83	1.398 (10)
C38—C43	1.395 (11)	C83—C84	1.375 (11)
C38—C39	1.394 (11)	C83—H83	0.9500
C39—C40	1.376 (11)	C84—C85	1.374 (11)
C39—H39	0.9500	C84—H84	0.9500
C40—C41	1.379 (11)	C85—C86	1.377 (10)
C40—H40	0.9500	C86—C87	1.366 (11)
C41—C42	1.397 (11)	C86—H86	0.9500
C42—C43	1.365 (11)	C87—H87	0.9500
C42—H42	0.9500	C88—H88A	0.9800
C43—H43	0.9500	C88—H88B	0.9800
C44—H44A	0.9800	C88—H88C	0.9800
			
C1—S1—C22	100.3 (4)	C45—S3—C66	99.4 (4)
C7—O1—C8	106.8 (6)	C51—O7—C52	105.6 (6)
C13—O2—C12	106.1 (6)	C56—O8—C57	105.2 (6)
C11—O3—C12	107.0 (6)	C58—O9—C57	106.5 (6)
C8—C1—C2	106.3 (7)	C52—C45—C46	108.5 (7)
C8—C1—S1	128.4 (6)	C52—C45—S3	127.8 (6)
C2—C1—S1	125.3 (6)	C46—C45—S3	123.6 (6)
C7—C2—C3	119.6 (8)	C47—C46—C51	119.3 (7)
C7—C2—C1	106.6 (7)	C47—C46—C45	136.6 (7)
C3—C2—C1	133.7 (8)	C51—C46—C45	104.0 (7)
C4—C3—C2	119.0 (7)	C46—C47—C48	119.8 (7)
C4—C3—H3	120.5	C46—C47—H47	120.1
C2—C3—H3	120.5	C48—C47—H47	120.1
C3—C4—C5	118.6 (7)	C47—C48—C49	118.6 (7)
C3—C4—C16	123.3 (7)	C47—C48—C60	122.2 (7)
C5—C4—C16	118.1 (7)	C49—C48—C60	119.1 (7)
C6—C5—C4	123.0 (8)	C50—C49—C48	122.0 (7)
C6—C5—H5	118.5	C50—C49—H49	119.0
C4—C5—H5	118.5	C48—C49—H49	119.0
C7—C6—C5	115.6 (8)	C49—C50—C51	117.7 (7)
C7—C6—H6	122.2	C49—C50—H50	121.2
C5—C6—H6	122.2	C51—C50—H50	121.2
C6—C7—O1	125.7 (7)	O7—C51—C50	126.1 (7)
C6—C7—C2	124.0 (8)	O7—C51—C46	111.4 (7)
O1—C7—C2	110.3 (7)	C50—C51—C46	122.6 (7)
C1—C8—O1	110.0 (7)	C45—C52—O7	110.4 (7)
C1—C8—C9	135.6 (7)	C45—C52—C53	137.6 (7)
O1—C8—C9	114.3 (7)	O7—C52—C53	111.8 (7)
C10—C9—C15	119.6 (7)	C54—C53—C59	119.0 (7)
C10—C9—C8	121.7 (7)	C54—C53—C52	120.5 (7)
C15—C9—C8	118.7 (7)	C59—C53—C52	120.5 (7)
C11—C10—C9	117.3 (8)	C55—C54—C53	121.8 (8)
C11—C10—H10	121.3	C55—C54—H54	119.1
C9—C10—H10	121.3	C53—C54—H54	119.1
C10—C11—C13	122.9 (8)	C56—C55—C54	117.3 (8)
C10—C11—O3	127.3 (8)	C56—C55—H55	121.4
C13—C11—O3	109.8 (7)	C54—C55—H55	121.4
O3—C12—O2	107.8 (7)	C55—C56—O8	128.0 (7)
O3—C12—H12A	110.2	C55—C56—C58	121.3 (7)
O2—C12—H12A	110.2	O8—C56—C58	110.6 (7)
O3—C12—H12B	110.2	O9—C57—O8	108.1 (6)
O2—C12—H12B	110.2	O9—C57—H57A	110.1
H12A—C12—H12B	108.5	O8—C57—H57A	110.1
C14—C13—C11	122.0 (8)	O9—C57—H57B	110.1
C14—C13—O2	128.7 (7)	O8—C57—H57B	110.1
C11—C13—O2	109.3 (7)	H57A—C57—H57B	108.4
C13—C14—C15	116.9 (8)	C59—C58—O9	128.5 (7)
C13—C14—H14	121.6	C59—C58—C56	122.7 (8)
C15—C14—H14	121.6	O9—C58—C56	108.9 (7)
C14—C15—C9	121.3 (8)	C58—C59—C53	117.9 (8)
C14—C15—H15	119.4	C58—C59—H59	121.0
C9—C15—H15	119.4	C53—C59—H59	121.0
C21—C16—C17	118.1 (8)	C65—C60—C61	116.7 (7)
C21—C16—C4	119.8 (7)	C65—C60—C48	122.7 (7)
C17—C16—C4	122.1 (7)	C61—C60—C48	120.6 (7)
C18—C17—C16	120.8 (8)	C62—C61—C60	121.2 (7)
C18—C17—H17	119.6	C62—C61—H61	119.4
C16—C17—H17	119.6	C60—C61—H61	119.4
C17—C18—C19	120.2 (8)	C61—C62—C63	120.5 (8)
C17—C18—H18	119.9	C61—C62—H62	119.8
C19—C18—H18	119.9	C63—C62—H62	119.8
C18—C19—C20	120.4 (8)	C62—C63—C64	119.8 (7)
C18—C19—Br1	120.8 (7)	C62—C63—Br3	120.2 (6)
C20—C19—Br1	118.7 (6)	C64—C63—Br3	120.0 (6)
C21—C20—C19	119.3 (8)	C65—C64—C63	119.9 (8)
C21—C20—H20	120.4	C65—C64—H64	120.1
C19—C20—H20	120.4	C63—C64—H64	120.1
C20—C21—C16	121.0 (8)	C64—C65—C60	121.9 (8)
C20—C21—H21	119.5	C64—C65—H65	119.1
C16—C21—H21	119.5	C60—C65—H65	119.1
S1—C22—H22A	109.5	S3—C66—H66A	109.5
S1—C22—H22B	109.5	S3—C66—H66B	109.5
H22A—C22—H22B	109.5	H66A—C66—H66B	109.5
S1—C22—H22C	109.5	S3—C66—H66C	109.5
H22A—C22—H22C	109.5	H66A—C66—H66C	109.5
H22B—C22—H22C	109.5	H66B—C66—H66C	109.5
C23—S2—C44	100.7 (4)	C67—S4—C88	100.3 (4)
C29—O4—C30	106.1 (6)	C73—O10—C74	105.6 (6)
C34—O5—C35	104.7 (6)	C80—O11—C79	105.7 (6)
C36—O6—C35	105.6 (6)	C78—O12—C79	105.6 (6)
C30—C23—C24	106.2 (7)	C74—C67—C68	107.5 (7)
C30—C23—S2	129.9 (6)	C74—C67—S4	129.2 (6)
C24—C23—S2	123.9 (6)	C68—C67—S4	123.3 (6)
C25—C24—C29	119.4 (8)	C69—C68—C73	120.0 (8)
C25—C24—C23	134.7 (8)	C69—C68—C67	135.7 (8)
C29—C24—C23	106.0 (7)	C73—C68—C67	104.1 (7)
C24—C25—C26	119.8 (8)	C68—C69—C70	119.0 (7)
C24—C25—H25	120.1	C68—C69—H69	120.5
C26—C25—H25	120.1	C70—C69—H69	120.5
C25—C26—C27	117.9 (8)	C69—C70—C71	119.2 (7)
C25—C26—C38	122.1 (7)	C69—C70—C82	122.7 (7)
C27—C26—C38	120.0 (7)	C71—C70—C82	118.1 (7)
C28—C27—C26	123.2 (8)	C72—C71—C70	122.4 (7)
C28—C27—H27	118.4	C72—C71—H71	118.8
C26—C27—H27	118.4	C70—C71—H71	118.8
C27—C28—C29	115.9 (8)	C71—C72—C73	116.5 (7)
C27—C28—H28	122.1	C71—C72—H72	121.8
C29—C28—H28	122.1	C73—C72—H72	121.8
O4—C29—C28	125.7 (7)	O10—C73—C68	112.3 (7)
O4—C29—C24	110.5 (7)	O10—C73—C72	124.8 (7)
C28—C29—C24	123.8 (8)	C68—C73—C72	123.0 (8)
C23—C30—O4	111.3 (7)	C67—C74—O10	110.4 (7)
C23—C30—C31	135.6 (8)	C67—C74—C75	136.4 (7)
O4—C30—C31	113.1 (7)	O10—C74—C75	113.2 (7)
C32—C31—C37	118.3 (8)	C76—C75—C81	119.6 (8)
C32—C31—C30	120.3 (7)	C76—C75—C74	118.9 (7)
C37—C31—C30	121.3 (7)	C81—C75—C74	121.5 (7)
C31—C32—C33	122.5 (8)	C77—C76—C75	122.0 (8)
C31—C32—H32	118.8	C77—C76—H76	119.0
C33—C32—H32	118.8	C75—C76—H76	119.0
C34—C33—C32	116.8 (8)	C78—C77—C76	116.5 (8)
C34—C33—H33	121.6	C78—C77—H77	121.7
C32—C33—H33	121.6	C76—C77—H77	121.7
C33—C34—C36	121.5 (8)	C77—C78—O12	127.9 (7)
C33—C34—O5	127.8 (7)	C77—C78—C80	122.3 (8)
C36—C34—O5	110.7 (7)	O12—C78—C80	109.7 (7)
O6—C35—O5	109.2 (7)	O12—C79—O11	107.0 (6)
O6—C35—H35A	109.8	O12—C79—H79A	110.3
O5—C35—H35A	109.8	O11—C79—H79A	110.3
O6—C35—H35B	109.8	O12—C79—H79B	110.3
O5—C35—H35B	109.8	O11—C79—H79B	110.3
H35A—C35—H35B	108.3	H79A—C79—H79B	108.6
C37—C36—C34	123.0 (8)	C81—C80—O11	127.9 (8)
C37—C36—O6	127.8 (7)	C81—C80—C78	122.6 (8)
C34—C36—O6	109.1 (7)	O11—C80—C78	109.5 (7)
C36—C37—C31	117.8 (8)	C80—C81—C75	117.0 (8)
C36—C37—H37	121.1	C80—C81—H81	121.5
C31—C37—H37	121.1	C75—C81—H81	121.5
C43—C38—C39	116.5 (7)	C87—C82—C83	117.0 (7)
C43—C38—C26	121.9 (7)	C87—C82—C70	121.6 (7)
C39—C38—C26	121.5 (7)	C83—C82—C70	121.4 (7)
C40—C39—C38	122.0 (8)	C84—C83—C82	121.9 (8)
C40—C39—H39	119.0	C84—C83—H83	119.1
C38—C39—H39	119.0	C82—C83—H83	119.1
C39—C40—C41	119.7 (8)	C83—C84—C85	118.8 (8)
C39—C40—H40	120.1	C83—C84—H84	120.6
C41—C40—H40	120.1	C85—C84—H84	120.6
C40—C41—C42	119.9 (8)	C84—C85—C86	121.3 (8)
C40—C41—Br2	120.3 (6)	C84—C85—Br4	119.6 (6)
C42—C41—Br2	119.9 (6)	C86—C85—Br4	119.1 (6)
C43—C42—C41	119.1 (8)	C87—C86—C85	119.5 (8)
C43—C42—H42	120.5	C87—C86—H86	120.3
C41—C42—H42	120.5	C85—C86—H86	120.3
C42—C43—C38	122.7 (8)	C86—C87—C82	121.5 (8)
C42—C43—H43	118.6	C86—C87—H87	119.3
C38—C43—H43	118.6	C82—C87—H87	119.3
S2—C44—H44A	109.5	S4—C88—H88A	109.5
S2—C44—H44B	109.5	S4—C88—H88B	109.5
H44A—C44—H44B	109.5	H88A—C88—H88B	109.5
S2—C44—H44C	109.5	S4—C88—H88C	109.5
H44A—C44—H44C	109.5	H88A—C88—H88C	109.5
H44B—C44—H44C	109.5	H88B—C88—H88C	109.5
			
C22—S1—C1—C8	−80.2 (8)	C66—S3—C45—C52	−96.5 (8)
C22—S1—C1—C2	102.2 (7)	C66—S3—C45—C46	85.5 (7)
C8—C1—C2—C7	0.5 (9)	C52—C45—C46—C47	178.3 (9)
S1—C1—C2—C7	178.5 (6)	S3—C45—C46—C47	−3.4 (14)
C8—C1—C2—C3	176.4 (8)	C52—C45—C46—C51	0.4 (9)
S1—C1—C2—C3	−5.6 (13)	S3—C45—C46—C51	178.8 (6)
C7—C2—C3—C4	−2.1 (12)	C51—C46—C47—C48	−1.1 (12)
C1—C2—C3—C4	−177.5 (8)	C45—C46—C47—C48	−178.7 (8)
C2—C3—C4—C5	−1.0 (11)	C46—C47—C48—C49	−1.1 (11)
C2—C3—C4—C16	−178.6 (7)	C46—C47—C48—C60	−178.0 (7)
C3—C4—C5—C6	3.3 (12)	C47—C48—C49—C50	2.1 (11)
C16—C4—C5—C6	−179.0 (7)	C60—C48—C49—C50	179.1 (7)
C4—C5—C6—C7	−2.3 (12)	C48—C49—C50—C51	−0.9 (12)
C5—C6—C7—O1	179.1 (7)	C52—O7—C51—C50	179.2 (8)
C5—C6—C7—C2	−1.0 (13)	C52—O7—C51—C46	0.6 (8)
C8—O1—C7—C6	179.9 (8)	C49—C50—C51—O7	−179.9 (7)
C8—O1—C7—C2	−0.1 (9)	C49—C50—C51—C46	−1.4 (12)
C3—C2—C7—C6	3.2 (13)	C47—C46—C51—O7	−178.9 (7)
C1—C2—C7—C6	179.8 (8)	C45—C46—C51—O7	−0.6 (9)
C3—C2—C7—O1	−176.8 (7)	C47—C46—C51—C50	2.4 (12)
C1—C2—C7—O1	−0.3 (9)	C45—C46—C51—C50	−179.3 (7)
C2—C1—C8—O1	−0.6 (9)	C46—C45—C52—O7	−0.1 (9)
S1—C1—C8—O1	−178.5 (5)	S3—C45—C52—O7	−178.4 (5)
C2—C1—C8—C9	−177.9 (9)	C46—C45—C52—C53	−173.8 (9)
S1—C1—C8—C9	4.2 (14)	S3—C45—C52—C53	7.9 (15)
C7—O1—C8—C1	0.4 (9)	C51—O7—C52—C45	−0.3 (8)
C7—O1—C8—C9	178.4 (7)	C51—O7—C52—C53	175.2 (6)
C1—C8—C9—C10	−15.1 (15)	C45—C52—C53—C54	157.6 (9)
O1—C8—C9—C10	167.6 (7)	O7—C52—C53—C54	−16.0 (10)
C1—C8—C9—C15	166.8 (9)	C45—C52—C53—C59	−21.9 (14)
O1—C8—C9—C15	−10.4 (11)	O7—C52—C53—C59	164.4 (7)
C15—C9—C10—C11	−1.0 (12)	C59—C53—C54—C55	−0.2 (12)
C8—C9—C10—C11	−179.1 (8)	C52—C53—C54—C55	−179.7 (7)
C9—C10—C11—C13	−0.3 (13)	C53—C54—C55—C56	0.7 (12)
C9—C10—C11—O3	180.0 (7)	C54—C55—C56—O8	−178.6 (8)
C12—O3—C11—C10	178.9 (9)	C54—C55—C56—C58	−1.0 (12)
C12—O3—C11—C13	−0.8 (9)	C57—O8—C56—C55	−177.2 (9)
C11—O3—C12—O2	1.6 (9)	C57—O8—C56—C58	5.0 (9)
C13—O2—C12—O3	−1.8 (9)	C58—O9—C57—O8	8.2 (10)
C10—C11—C13—C14	0.2 (13)	C56—O8—C57—O9	−8.1 (10)
O3—C11—C13—C14	179.9 (7)	C57—O9—C58—C59	176.1 (9)
C10—C11—C13—O2	180.0 (8)	C57—O9—C58—C56	−5.2 (9)
O3—C11—C13—O2	−0.3 (9)	C55—C56—C58—C59	0.8 (13)
C12—O2—C13—C14	−179.0 (9)	O8—C56—C58—C59	178.9 (8)
C12—O2—C13—C11	1.3 (9)	C55—C56—C58—O9	−178.0 (7)
C11—C13—C14—C15	1.3 (12)	O8—C56—C58—O9	0.1 (9)
O2—C13—C14—C15	−178.5 (8)	O9—C58—C59—C53	178.2 (7)
C13—C14—C15—C9	−2.6 (12)	C56—C58—C59—C53	−0.3 (12)
C10—C9—C15—C14	2.6 (12)	C54—C53—C59—C58	0.0 (12)
C8—C9—C15—C14	−179.3 (8)	C52—C53—C59—C58	179.5 (7)
C3—C4—C16—C21	19.7 (12)	C47—C48—C60—C65	22.7 (12)
C5—C4—C16—C21	−157.9 (7)	C49—C48—C60—C65	−154.2 (7)
C3—C4—C16—C17	−158.3 (8)	C47—C48—C60—C61	−158.1 (8)
C5—C4—C16—C17	24.1 (11)	C49—C48—C60—C61	25.0 (11)
C21—C16—C17—C18	4.9 (12)	C65—C60—C61—C62	2.9 (12)
C4—C16—C17—C18	−177.1 (8)	C48—C60—C61—C62	−176.3 (8)
C16—C17—C18—C19	−3.3 (13)	C60—C61—C62—C63	−1.8 (13)
C17—C18—C19—C20	−0.8 (13)	C61—C62—C63—C64	−0.5 (13)
C17—C18—C19—Br1	177.6 (6)	C61—C62—C63—Br3	177.1 (7)
C18—C19—C20—C21	3.2 (13)	C62—C63—C64—C65	1.6 (13)
Br1—C19—C20—C21	−175.2 (6)	Br3—C63—C64—C65	−176.0 (6)
C19—C20—C21—C16	−1.6 (12)	C63—C64—C65—C60	−0.4 (13)
C17—C16—C21—C20	−2.4 (12)	C61—C60—C65—C64	−1.8 (12)
C4—C16—C21—C20	179.5 (7)	C48—C60—C65—C64	177.4 (7)
C44—S2—C23—C30	90.9 (9)	C88—S4—C67—C74	−86.5 (8)
C44—S2—C23—C24	−91.2 (7)	C88—S4—C67—C68	95.1 (7)
C30—C23—C24—C25	−179.7 (9)	C74—C67—C68—C69	173.8 (9)
S2—C23—C24—C25	2.0 (14)	S4—C67—C68—C69	−7.5 (13)
C30—C23—C24—C29	0.5 (9)	C74—C67—C68—C73	0.0 (9)
S2—C23—C24—C29	−177.8 (6)	S4—C67—C68—C73	178.7 (6)
C29—C24—C25—C26	0.3 (12)	C73—C68—C69—C70	−3.3 (11)
C23—C24—C25—C26	−179.4 (8)	C67—C68—C69—C70	−176.4 (8)
C24—C25—C26—C27	1.2 (12)	C68—C69—C70—C71	2.0 (11)
C24—C25—C26—C38	−178.8 (7)	C68—C69—C70—C82	−178.0 (7)
C25—C26—C27—C28	−2.0 (12)	C69—C70—C71—C72	−0.3 (12)
C38—C26—C27—C28	177.9 (8)	C82—C70—C71—C72	179.8 (7)
C26—C27—C28—C29	1.3 (13)	C70—C71—C72—C73	−0.1 (12)
C30—O4—C29—C28	−178.6 (8)	C74—O10—C73—C68	2.2 (9)
C30—O4—C29—C24	0.8 (9)	C74—O10—C73—C72	−177.2 (7)
C27—C28—C29—O4	179.8 (8)	C69—C68—C73—O10	−176.4 (7)
C27—C28—C29—C24	0.4 (12)	C67—C68—C73—O10	−1.4 (9)
C25—C24—C29—O4	179.3 (7)	C69—C68—C73—C72	3.0 (12)
C23—C24—C29—O4	−0.9 (9)	C67—C68—C73—C72	178.0 (7)
C25—C24—C29—C28	−1.2 (13)	C71—C72—C73—O10	178.1 (7)
C23—C24—C29—C28	178.6 (7)	C71—C72—C73—C68	−1.2 (12)
C24—C23—C30—O4	0.0 (9)	C68—C67—C74—O10	1.3 (9)
S2—C23—C30—O4	178.1 (6)	S4—C67—C74—O10	−177.3 (6)
C24—C23—C30—C31	178.3 (9)	C68—C67—C74—C75	−179.0 (9)
S2—C23—C30—C31	−3.5 (15)	S4—C67—C74—C75	2.5 (15)
C29—O4—C30—C23	−0.5 (9)	C73—O10—C74—C67	−2.1 (8)
C29—O4—C30—C31	−179.3 (7)	C73—O10—C74—C75	178.1 (6)
C23—C30—C31—C32	−173.2 (9)	C67—C74—C75—C76	165.1 (9)
O4—C30—C31—C32	5.2 (11)	O10—C74—C75—C76	−15.2 (11)
C23—C30—C31—C37	9.0 (15)	C67—C74—C75—C81	−14.0 (15)
O4—C30—C31—C37	−172.6 (7)	O10—C74—C75—C81	165.7 (7)
C37—C31—C32—C33	−0.4 (12)	C81—C75—C76—C77	−0.9 (12)
C30—C31—C32—C33	−178.3 (8)	C74—C75—C76—C77	180.0 (8)
C31—C32—C33—C34	−1.0 (13)	C75—C76—C77—C78	1.7 (13)
C32—C33—C34—C36	1.6 (13)	C76—C77—C78—O12	−177.4 (8)
C32—C33—C34—O5	177.8 (8)	C76—C77—C78—C80	−1.9 (13)
C35—O5—C34—C33	177.0 (9)	C79—O12—C78—C77	−173.5 (9)
C35—O5—C34—C36	−6.4 (9)	C79—O12—C78—C80	10.6 (9)
C36—O6—C35—O5	−8.3 (9)	C78—O12—C79—O11	−15.5 (9)
C34—O5—C35—O6	9.1 (9)	C80—O11—C79—O12	14.7 (9)
C33—C34—C36—C37	−1.0 (13)	C79—O11—C80—C81	172.8 (9)
O5—C34—C36—C37	−177.8 (7)	C79—O11—C80—C78	−8.4 (9)
C33—C34—C36—O6	178.2 (7)	C77—C78—C80—C81	1.3 (13)
O5—C34—C36—O6	1.4 (9)	O12—C78—C80—C81	177.5 (7)
C35—O6—C36—C37	−176.6 (9)	C77—C78—C80—O11	−177.6 (7)
C35—O6—C36—C34	4.3 (9)	O12—C78—C80—O11	−1.4 (9)
C34—C36—C37—C31	−0.4 (13)	O11—C80—C81—C75	178.3 (7)
O6—C36—C37—C31	−179.4 (7)	C78—C80—C81—C75	−0.3 (12)
C32—C31—C37—C36	1.1 (12)	C76—C75—C81—C80	0.2 (12)
C30—C31—C37—C36	178.9 (8)	C74—C75—C81—C80	179.3 (7)
C25—C26—C38—C43	160.9 (8)	C69—C70—C82—C87	24.2 (12)
C27—C26—C38—C43	−19.1 (12)	C71—C70—C82—C87	−155.9 (7)
C25—C26—C38—C39	−18.0 (12)	C69—C70—C82—C83	−157.2 (8)
C27—C26—C38—C39	162.0 (8)	C71—C70—C82—C83	22.7 (11)
C43—C38—C39—C40	2.1 (12)	C87—C82—C83—C84	2.2 (12)
C26—C38—C39—C40	−179.0 (7)	C70—C82—C83—C84	−176.5 (8)
C38—C39—C40—C41	0.0 (12)	C82—C83—C84—C85	1.3 (13)
C39—C40—C41—C42	−1.8 (12)	C83—C84—C85—C86	−3.9 (13)
C39—C40—C41—Br2	178.6 (6)	C83—C84—C85—Br4	175.5 (6)
C40—C41—C42—C43	1.3 (13)	C84—C85—C86—C87	3.0 (13)
Br2—C41—C42—C43	−179.0 (6)	Br4—C85—C86—C87	−176.5 (6)
C41—C42—C43—C38	0.9 (13)	C85—C86—C87—C82	0.7 (12)
C39—C38—C43—C42	−2.5 (12)	C83—C82—C87—C86	−3.2 (12)
C26—C38—C43—C42	178.5 (8)	C70—C82—C87—C86	175.5 (7)

### Hydrogen-bond geometry (Å, °)

**Table:**

*D*—H···*A*	*D*—H	H···*A*	*D*···*A*	*D*—H···*A*
C12—H12A···O4	0.99	2.58	3.50 (1)	154
C21—H21···O6^i^	0.95	2.51	3.37 (1)	150
C57—H57A···O10^ii^	0.99	2.50	3.32 (1)	141
C65—H65···O11^iii^	0.95	2.58	3.47 (1)	156
C79—H79B···O7^iv^	0.99	2.56	3.26 (1)	128

Symmetry codes: (i) −*x*+2, −*y*+1, −*z*; (ii) −*x*+1, −*y*+1, −*z*+1; (iii) *x*, *y*+1, *z*; (iv) −*x*, −*y*+1, −*z*+1.
